# Gas plasma-spurred wound healing is accompanied by regulation of focal adhesion, matrix remodeling, and tissue oxygenation

**DOI:** 10.1016/j.redox.2020.101809

**Published:** 2020-11-25

**Authors:** Anke Schmidt, Grit Liebelt, Felix Nießner, Thomas von Woedtke, Sander Bekeschus

**Affiliations:** a ZIK Plasmatis, Leibniz Institute for Plasma Science and Technology (INP), Felix-Hausdorff-Str. 2, 17489, Greifswald, Germany; b Department of Hygiene and Environmental Medicine, Greifswald University Medical Center, Ferdinand-Sauerbruch-Str., 17475, Greifswald, Germany

**Keywords:** Dermal fibroblasts, Extracellular matrix, Hyperspectral imaging, Plasma medicine, Reactive oxygen and nitrogen species

## Abstract

In response to injury, efficient migration of skin cells to rapidly close the wound and restore barrier function requires a range of coordinated processes in cell spreading and migration. Gas plasma technology produces therapeutic reactive species that promote skin regeneration by driving proliferation and angiogenesis. However, the underlying molecular mechanisms regulating gas plasma-aided cell adhesion and matrix remodeling essential for wound closure remain elusive. Here, we combined *in vitro* analyses in primary dermal fibroblasts isolated from murine skin with *in vivo* studies in a murine wound model to demonstrate that gas plasma treatment changed phosphorylation of signaling molecules such as focal adhesion kinase and paxillin α in adhesion-associated complexes. In addition to cell spreading and migration, gas plasma exposure affected cell surface adhesion receptors (e.g., integrinα5β1, syndecan 4), structural proteins (e.g., vinculin, talin, actin), and transcription of genes associated with differentiation markers of fibroblasts-to-myofibroblasts and epithelial-to-mesenchymal transition, cellular protrusions, fibronectin fibrillogenesis, matrix metabolism, and matrix metalloproteinase activity. Finally, we documented that gas plasma exposure increased tissue oxygenation and skin perfusion during ROS-driven wound healing. Altogether, these results provide critical insights into the molecular machinery of gas plasma-assisted wound healing mechanisms.

## Graphical abstract

Other constituents of focal adhesions participate in the structural link between membrane receptors and the actin cytoskeleton [1]. A wound healing phase-dependent action of intracellular proteases (MMPs) senses extracellular signals after gas plasma treatment by modifying the activation state of structural proteins (e.g., vinculin and talin), which are associated with actin and integrins.

**Figure:**
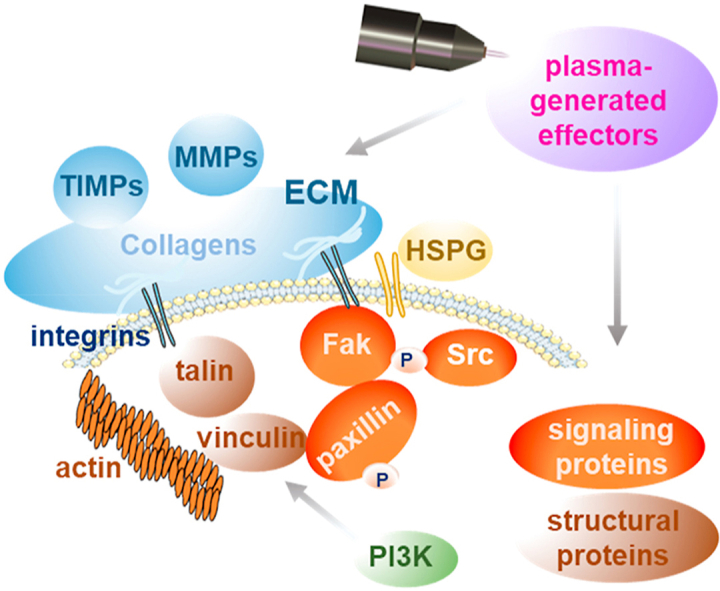

## 1 Introduction

Reactive oxygen and nitrogen species (here summarized as ROS) play a role in redox-mediated wound healing processes and skin tissue regeneration [2,3]. ROS signaling mediates distinct pathways in regenerating tissue, e.g., apoptosis and c-Jun N-terminal kinase activation [4]. ROS as signalizing molecules change gene and protein expression of extracellular matrix (ECM) components as well as cytoskeletal and adhesion proteins, resulting in the fine-tuned regulation of collagen degradation via matrix metalloproteinases (MMPs) and their inhibitors (TIMPs) [5,6]. Intracellularly generated ROS are essential mediators of cell adhesion by acting on integrin and growth factor signaling pathways [7].

Integrin adhesions in cultured cells are subdivided into different complexes with distinct molecular compositions and functions [8]. Focal complexes (FX) are transformed into focal adhesions (FA) within minutes [9] and can develop into fibrillary adhesions (FB) [10]. As specialized adhesive structures, these complexes serve together with heparan sulfate proteoglycans (HSPG) [11] as communication units between adjacent cells and ECM for mediating cell adhesion. All types of cell surface-adhesion complexes are involved in cytoskeletal organization and signaling pathways [12] and are essential in maintaining attachments to ECM through a subset of proteins such as focal adhesion kinase, paxillin, actin, vinculin, and talin [13]. Degradation, deposition, and disruption of ECM components and collagen fiber synthesis [14], as well as dysregulation of MMPs and TIMPs, are characteristics of non-healing chronic wounds. Other adverse events in chronic wounds are alterations in integrin-mediated adhesion and migration, dysregulated fibroblasts-to-myofibroblasts (FMT) and epithelial-to-mesenchymal transition (EMT), and loss of elasticity of connective skin tissue along with dermal-junctional connections [15]. FMT and EMT are major factors of fibrotic processes [16] mechanistically associated with changes in the structural integrity of dermal skin layers and attenuated *de novo* formation of α smooth muscle actin (αSma) fibers [17]. However, the role of ROS in these processes is underexplored.

The ROS-generating nature of cold physical plasma, a partially ionized gas, alters cellular responses critical in skin regeneration [18]. As such, gas plasma was found to support physiological wound healing [19] via exogenous exposure to therapeutic doses of ROS and subsequent ROS-induced signaling pathways, potentially driving paracrine effects [20]. Gas plasma-generated ROS such as superoxide radicals and nitric oxide [21] affect cell proliferation, migration, chemotaxis, apoptosis, and inflammatory gene and protein expression [18,22,23]. However, many factors are associated with tissue repair, which might contribute to the fact that chronic wounds still are a clinical problem. Gas plasma treatment promotes the healing of chronic wounds [24] and is an integraded part of applied medicine at many dermatological centers in central Europe [25], but the mechanisms of action remain elusive.

To this end, we studied the gas plasma-induced cellular responses in wound-relevant cells and wounds on a molecular level concerning integrin-adhesion complexes, modulation of adhesion sites, and interaction with matrix proteins such as collagens and vinculin. The aim was to test how therapeutic ROS delivery may trigger appropriate motility signaling via integrins for mobilizing the migration of fibroblasts into the wound. Migration processes involve actin re-organization and changes in focal adhesion sites, cellular protrusions, and MMP expression with subsequent matrix degradation, and we extended our study on these aspects. We identified changes in cellular adhesiveness, MMP activity, and tissue oxygenation in superficial skin layers, presumably acting in concert in gas plasma-promoted wound healing.

## 2 Materials and methods

### 2.1 In vivo model and gas plasma treatment

SKH1-hr hairless immunocompetent mice (Charles River Laboratories, Sulzfeld, Germany) were housed under standard conditions in the animal facilities of the Medical Faculty of the University of Rostock, Germany, and Institute of Pharmacology of the University of Greifswald, Germany. Study approval by the regional ethical board (approval numbers 7221.3-1-013/14 and 7221.3-044/16) was obtained. Experimental details of the dermal full-thickness ear wound model have been described previously [19,[26], [27], [28]]. Briefly, ear wounds were created by removing upper skin layers. The atmospheric pressure argon plasma jet kINPen (neoplas MED, Greifswald, Germany) was used, which ionizes a flow (5 standard liters per minute) of argon gas (Air Liquide, Krefeld, Germany) at 1 MHz. Ear wounds were gas plasma-treated (3 s) three times per week over 14 consecutive days or were left untreated (n ≥ 9/group). Assays on wound tissues were performed on day 6 (d6, after three times of gas plasma treatments in total) and day 15 (d15, after six times in total, n ≥ 9/group).

### 2.2 pDF preparation, culture, and gas plasma treatment

Primary dermal fibroblasts (pDFs) and keratinocytes were isolated from skins of a total of 10 healthy SKH1 mice by enzyme-mediated removal of the epidermal layer and digestion of the dermis according to recommendations of an epidermis dissociation kit. The cell suspension was homogenized in gentleMACS C tubes using an OctaMACS dissociator (Miltenyi Biotec, Bergisch-Gladbach, Germany) to obtain live cells, and a FastPrep-24 5G homogenizer (MP biomedicals, Heidelberg, Germany) for obtaining lysates. For live cells, the suspension was passed through 70 μm MACS SmartStrainers (Miltenyi) to separate individual cells. pDFs were cultured over ten days in fibroblast Eagle's minimal essential medium EMEM medium (PromoCell, Heidelberg, Germany) supplemented with 10% fetal bovine serum, 0.1 mg/ml penicillin/streptomycin, and 2 mM glutamine (Sigma-Aldrich, Taufkirchen, Germany) in a humidified incubator at 37 °C with 5% CO_2_. In experiments, early passages from 1 to 5 were used, and the cell culture medium was changed every 2–3 days. pDFs were indirectly gas plasma-treated by exposing 5 ml of fully supplemented Roswell Park Memorial Institute (RPMI) for short (20 s), intermediate (60 s), and long (180 s) durations at a distance of 9 mm using a computer-controlled *xyz*-table (CNC step, Germany). The isolated pDFs were incubated in untreated or gas plasma-treated medium for 2 h, followed by the addition of fresh medium. Sample analysis was done after a total of 2 h, 6 h, and 24 h. In some experiments, 0.5 h of incubation was used. Results from negative placebo-treated (argon) controls were not different from those of untreated controls (data not shown).

### 2.3 Analysis of cell viability, proliferation, and motility

pDFs were analyzed via live/dead and redox-based assays as described before [29]. Briefly, pDFs were stained with 2 μM of Calcein-AM, 1 μM of propidium iodide (PI), and 5 μM of Hoechst 33342 (Life Technologies, Carlsbad, CA, USA), and imaged using fluorescence microscopy (Axio Observer Z.1; Zeiss, Jena, Germany). The redox-based resazurin (7-hydroxy-3H-phenoxazin-3-one 10-oxide) proliferation assay of 3.5 × 10^3^ initially seeded pDFs was determined using a multimode plate reader (F200; Tecan, Männedorf, Switzerland) at *λ*_ex_ 560 nm and *λ*_em_ 590 nm. The motility of pDFs was determined in 96-well plates using the scratch assay 24 h after reaching confluence. After scratching, the cells were washed, incubated with the gas plasma-treated medium, and imaged (brightfield).

### 2.4 mRNA and protein quantification

For mRNA analysis on d6 and d15, ear wound regions were collected, and homogenization of wound tissue was performed using a FastPrep-24 5G homogenizer (MP biomedicals, Heidelberg, Germany). After lysis of pDFs and skin tissue in RNA lysis buffer, total RNA was isolated according to the manufacturer's instructions (Bio&Sell, Feucht, Germany), and mRNA expression levels were determined by quantitative PCR (qPCR). Briefly, 1 μg of RNA was transcribed into cDNA, and qPCR was conducted in duplicates using SYBR Green I Master (Roche Diagnostics, Basel, Switzerland). Gene-specific primers (Table S1) were used from BioTez (Berlin, Germany). The housekeeping gene *GAPDH*, whose expression was unaffected by gas plasma treatment, was used as an internal normalization control. Gene expression was analyzed using the _ΔΔ_CT method.

For protein analysis, tissue and pDFs were lysed in RIPA buffer containing protease and phosphatase inhibitors (cOmplete Mini, phosSTOP, PMSF; Sigma-Aldrich). Protein expression levels of the focal adhesion kinase (Fak), paxillin α (Pxnα), vinculin (Vcl), their phosphorylated counterparts (pFak, pPxn, pVcl), tyrosine-protein kinase (Src), phosphoinositide 3-kinase (PI3K), and collagen (Col) were determined using corresponding antibodies (Cell signaling, Frankfurt, Germany). GAPDH served as housekeeping control. The WES system (ProteinSimple, San Jose, California, USA) was used for quantification according to the manufacturer's instructions and Compass software (ProteinSimple), and expressed as fold change compared to the corresponding control.

### 2.5 Immunofluorescence imaging

pDFs were seeded on glass coverslips and exposed to gas plasma-treated medium (20 s) 24 h later. For immunofluorescence microscopy, cells were fixed in 4% paraformaldehyde (PFA, Sigma-Aldrich) for 20 min, washed, and permeabilized with Triton X-100 (0.01% in PBS). On d6 and d15, ear wound regions were collected and fixed in 4% PFA overnight and paraffinized. Paraffin blocks were cut into 5 μm-sections using a microtome, and tissue sections were stained with hematoxylin and eosin (H&E; Carl-Roth, Karlsruhe, Germany). Fixed pDFs and tissue sections were incubated with primary antibodies: anti-Vcl, anti-pVcl, anti-Fak, anti-pY397Fak, anti-Pxnα, and anti-pY118-Pxnα (all Cell Signaling), followed by a secondary antibody coupled to an Alexa-Fluor 594 dye (Life Technologies). Collagen fibers were stained with picrosirius red (Direktrot 80; Sigma-Aldrich). 4′,6-Diamidin-2-phenylindole (DAPI, final concentration 1 μM; BioLegend, UK) were used to counterstain nuclei*.* Stained pDFs were mounted onto glass microscope slides using mounting medium (VectaShield; Biozol, Eching, Germany) prior to fluorescence microscopy using an Axio Observer Z.1. For image analysis, pDFs were counted, and the ratio between green or red nuclei over the total number of nuclei (blue) in a minimum of three fields of view per sample was calculated using ImageJ.

The tissue sections were then incubated with primary anti-pY397Fak targeted against phosphorylated Fak (1:100) for 3 h at 37 °C in a humid chamber. After washing, peroxidase-conjugated secondary anti-rabbit IgG (Sigma, Steinheim, Germany) was added for 1 h at room temperature, followed by their chromogenic detection using 3,3′-diaminobenzidine (DAB; Sigma, Steinheim, Germany). The slides were counterstained with Harris's hematoxylin, dehydrated, and mounted with mounting medium (VectaShield).

### 2.6 Measurement of gelatinase activity

pDFs were grown in FBS-free medium for 24 h, and supernatants of gas plasma-treated pDFs and their untreated controls were collected, centrifuged, and stored until use at −80 °C. The activity of the secreted gelatinase enzymes MMP2 and MMP9 was assessed by gel electrophoresis zymography using 10% (Novex) gelatin gel according to the vendor's protocol (Invitrogen, Carlsbad, CA, USA). Briefly, samples were mixed with 1% SDS buffer, electrophoresed, and washed after 30 min with renaturing and incubating buffers. Gels were stained with Coomassie dye (0.1%) for 1 h and de-stained in 7% acetic acid. Images were acquired, and the activity (active MMP2 with 62 kD; MMP9 with 82 kDa) was determined using densitometry analysis [30]. The activity of gelatinases during wound healing in our mouse model was determined using *in situ* zymography by dye-quenched (DQ) gelatin (Invitrogen, Carlsbad, CA, USA). Briefly, DQ-gelatin was added to the tissue sections, incubated at 37 °C for 2 h in the dark in a humid chamber, and washed three times in PBS. The signaling intensity of DQ-gelatin was scored using a histological score system between 1 and 10. Additional samples were incubated with an anti-MMP2 antibody (Cell Signaling) followed by incubation with a secondary Alexa-Fluor 594-labeled antibody (Life Technologies). Gelatinase activity was inhibited by the serine proteinase inhibitor phenylmethylsulfonyl fluoride (PMSF) and 1,10-phenanthroline monohydrate (Phe; both Sigma-Aldrich). Tissue sections were stained with DAPI and analyzed using fluorescence microscopy.

### 2.7 Measurement of microcirculation during wound healing

After anesthesia using ketamine and xylazine, the hyperspectral camera system TIVITA (Diaspective Vision, Wismar, Germany) was utilized for the acquisition of the tissue oxygenation (StO_2_, in %), near-infrared perfusion (NIR), tissue hemoglobin index (THI), and tissue water index (TWI) parameters. The working distance was 50 cm, and the spectral measurements were in the range between 500 and 1000 nm under standardized conditions (such as temperature and light). The measurements started with a baseline analysis on d0 before gas plasma treatment to assess the microcirculation parameter at baseline in untreated tissue. In untreated controls and 10 min after gas plasma treatment (3 s), wound oxygenation parameters were recorded from both ears and compared as indicated. The camera-specific software TIVITA Suite was used to calculate parameters in the circular areas in wound regions (pink line), blood vessels (purple line), and ear background (grey line). All measurements were redone every 3rd day up to the endpoint (d15).

### 2.8 Statistical analysis

Data are presented as mean ± S.E. of at least three independent experiments. Graphing and statistical analysis were performed using prism software 7.04 (GraphPad Software, San Diego, California, USA) and one-way analysis of variance (ANOVA) or *Student*'s t-test with *p*-values indicated by *p < 0.05, **p < 0.01, and ***p < 0.001.

## 3 Results

Extended gas plasma treatment decreased fibroblasts’ viability but promoted mobility by regulation of transmembrane receptors and actin cytoskeleton.

**Fig. 1:**
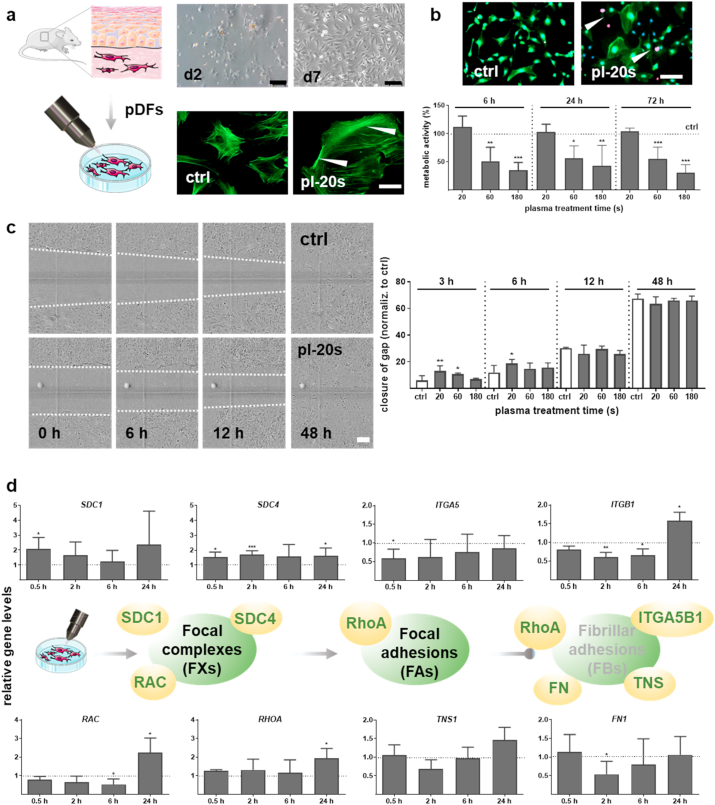
Gas plasma affected the viability, metabolic activity, and motility of dermal fibroblasts and regulated the molecular machinery of integrin adhesions. (a) Experimental workflow. Primary dermal fibroblasts (pFDs) were isolated from the skin of SKH1 mice and cultivated over ten days in fibroblast medium. pDFs were incubated with medium exposed to gas plasma for 20 s, 60 s, or 180 s, or were left untreated (ctrl), and analyzed by several down-stream assays. Representative phase-contrast images of the progression of pDFs after isolation from the skin of SKH1 mice on d2 and d7. Scale bars are 100 μm. F-actin stress fibers were visualized using FITC-phalloidin fluorescent probe (green); arrowheads show cell border staining in untreated (ctrl) and gas plasma-treated cells (pl-20 s). Scale bar is 50 μm (b) Live/dead assay measured at 6 h after gas plasma treatment, demonstrating mainly viable cells (green, calcein-positive) along with a few dead cells (red, propidium iodide-positive, pl-20 s) in comparison to control (ctrl). Scale bar is 50 μm. Quantification of metabolic activity of pDFs after 6 h, 24 h, and 72 h following gas plasma treatment. (c) Representative images showing migration of pDFs in untreated and gas plasma-treated (pl-20 s) pDFs. Scale bar is 200 μm. Quantification of wound closure and migratory behavior of pDFs up to 48 h after gas plasma treatment measured by wound healing scratch assay. (d) pDFs were harvested and lysed at 0.5 h, 2 h, 6 h, and 24 h after gas plasma treatment (20 s). qPCR of syndecans (*SDC1/4*), integrins (*ITGA5*, *ITGB1*), small GTP binding proteins (*RAC, RHOA),* tensin 1 *(TNS*), and fibronectin 1 (FN1) was perfomed in pDFs. Statistical analysis was done by unpaired two-tailed *Student*'s t-test with significances of *p < 0.05, **p < 0.01, and ***p < 0.001. (For interpretation of the references to color in this figure legend, the reader is referred to the Web version of this article.)

Fibroblasts are integrated into the dermal layer interacting with the extracellular matrix (ECM) and essential components in wound healing previously found to be spurred using gas plasma treatment in patients [24]. Both fibroblasts and their link into the ECM following plasma treatment is unexplored. Freshly isolated dermal fibroblasts (pDFs) were harvested from the healthy murine skin up to ten days (upper images, Fig. 1a). A more diffuse intracellular staining, and stronger staining at cell-cell contacts indicative of peripheral stress together with increased cell size and spreading was found after gas plasma treatment as identified via immunofluorescence staining of the cytoskeletal actin filaments (F-actin, lower images, Fig. 1a). Live (green)/dead (red) cell assay using calcein-AM and propidium iodide (PI) staining revealed mainly viable cells showed no marked changes in pDFs with 20 s of gas plasma treatment (images in Fig. 1b). For longer gas plasma treatment times, however, a significantly reduced viability of pDFs was observed (Fig. 1b).

Fibroblast motility is critical for migrating into the wound bed, so we used the scratch assay to assess this parameter (images in Fig. 1c). Gas plasma treatment spurred pDFs' motility at 3 h and 6 h post-exposure, while no changes were observed at later time points (Fig. 1c). This indicated a stimulatory effect of 20 s of gas plasma treatment, and this exposure time was used for subsequent experiments. Transmembrane receptors such as integrins (ITG) and heparan sulfate proteoglycans (HPSG), including syndecans (SDC), are tightly linked to motility. Using qPCR, we found *SDC1* and *SDC4* to be increased following gas plasma treatment, while *ITGA5* and *ITGB1* were decreased at shorter incubation times (upper diagrams, Fig. 1d). By contrast, *ITGA2* (CD49b), *ITGA1* (CD49a), and *ITGA6* (CD49f) remained unchanged up to 6 h post gas plasma exposure, with a significant increase of the latter at 24 h (Fig. S1a). Integrin adhesion complexes are formed by a network of signaling and scaffolding proteins involving small GTPase family members [8]. The expression of ras-related C3 botulinum toxin substrate *RAC* was significantly down-regulated at early time points after gas plasma treatment, whereas ras-homolog gene family member *RHOA* was upregulated in pDFs at 24 h. This suggested a transformation of focal complexes (FXs) into focal adhesions (FAs).

The development of FAs into fibrillary adhesions (FBs) is connected to the overexpression of the fibronectin receptor integrinα5β1 (ITGA5B1*)* and tensin 1 (TNS1), a marker of FBs. However, we observed a transient down-regulation of the *TNS1* and the pericellular fibronectin matrix, as shown by the quantification of fibronectin 1 (*FN1*), indicating changes in the adhesive phenotype with the presence of FAs but not FBs (lower diagrams, Fig. 1d). These results underlined that gas plasma treatment changes the formation and phenotype of integrin adhesions and migratory capacity of pDFs as shown in the schematic illustrations (Fig. 1d).

Gas plasma exposure affected the balance between integrin adhesion complexes by regulation of signaling and scaffolding molecules.

**Fig. 2:**
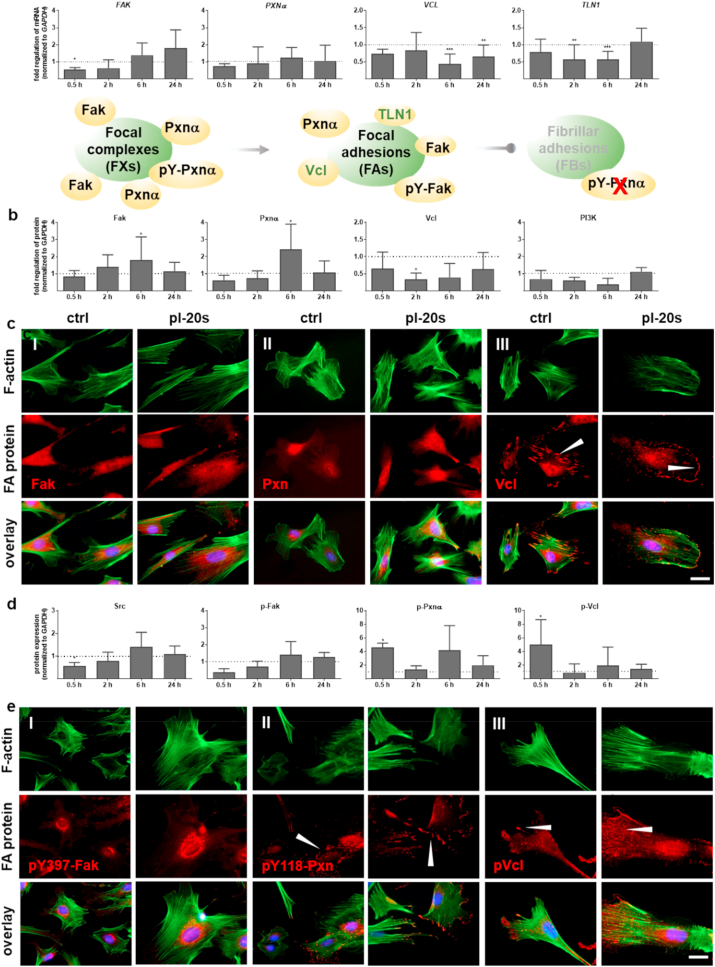
Gas plasma treatment modulated signaling and scaffolding proteins that link the integrin receptors to the actin cytoskeleton. (a) Gene expression levels of focal adhesion kinase (*FAK*), paxillin α (*PXNα*), vinculin (*VCL*), and talin (*TLN1*) were determined by qPCR. Scheme of focal adhesion complexes with associated protein targets. (b, d) Protein quantification of the non-phosphorylated proteins and activated pFak, pPxn, pVcl as well as tyrosine kinase (Src) and phosphoinositide-3-kinase (PI3K) were determined by Western blot analysis in relation to incubation time with gas plasma-treated medium as indicated. Data were normalized to *GAPDH* and untreated controls (ctrl) at indicated time points (n > 5) and presented as mean +SE. Statistical analysis was done by unpaired two-tailed *Student*'s t-test with significances of *p < 0.05, **p < 0.01, and ***p < 0.001. (c, e) pDFs were grown on glass coverslips, incubated with gas plasma-treated medium (pl-20 s), fixed, and subjected to fluorescent staining for determination of expression and distribution of FA proteins (red) such as anti-Fak (I), anti-Pxn (II), anti-Vcl (III, arrowheads show vinculin positive cellular protrusions), and their phosphorylated counterparts in (e) with anti-pY397-FAK (I), anti-p-Pxn (II), and anti-pVcl (III). The cell nuclei were stained with DAPI (blue). Scale bars are 50 μm. (For interpretation of the references to color in this figure legend, the reader is referred to the Web version of this article.)

Cellular migration, as a sequence of repetitive steps, includes the assembly and disassembly of (new) cell-matrix contacts [31] and the disassembly of cellular adhesions by the action of intracellular proteases and kinases at the integrin adhesion complexes (schemes in Fig. 2a). We found a similar gene expression and protein expression of several adhesion-associated signaling and structural proteins by qPCR (Fig. 2a) and Western blotting (Fig. 2b). Gas plasma treatment transiently decreased FA kinase (*FAK/*Fak) and paxillin α (*PXNα*) expression after 30 min, but continuously increased. Findings on the protein level were mostly congruent. Moreover, we demonstrated that gas plasma treatment significantly decreased vinculin (*VCL*/Vcl) expression, a protein involved in cell motility and adherence. As a critical cytoplasmic protein mediating integrin adhesion to the ECM, we determined the mRNA levels of talin 1 (*TLN1*), which was also down-regulated up to 6 h in gas plasma-treated pDFs. The phosphoinositide 3 kinase (PI3K) pathway is essential for redox regulation and might have been linked to these observations, but PI3K expression was unaltered after gas plasma treatment (Fig. 2b). Nevertheless, the intensity of Fak and Pxn was slightly increased as shown by fluorescence microscopy at 6 h suggested (Fig. 2c). The structural adapter protein vinculin interconnecting signals in FAs regulating integrin dynamics [32] and actin network [33] was found to stain stronger at the leading-edge lamellipodia and filopodia in gas plasma-treated cells (arrowhead, Fig. 2cIII). This indicated a gas plasma-supporting effect on the migratory ability of pDFs.

Tyrosine phosphorylation of Fak and Pxnα follows integrin activation and growth factor simulation [8]. Therefore, we quantified the expression pattern of the tyrosine protein kinase Src showing a significantly down-regulation on early time points following gas plasma treatment. Accordingly, the expression level of activated pY397-FAK protein was slightly decreased early (0.5 h) at the protein level (Fig. 2d). Next, we validated a moderate to significant phosphorylation of Pxnα (II) and Vcl (III) in response to gas plasma treatment after short incubation times (Fig. 2d–e), indicative of enhanced migration [34,35]. Moreover, phosphorylated paxillin is found mostly in focal complexes (FXs) and FAs but not in fibrillary adhesions (FBs), validating the absence of FBs in gas plasma-treated dermal fibroblasts.

A regulator of the stress response is the heat shock transcription factor HSF1, which affects the ability of cells to migrate and spread via transcriptional regulation of vinculin [36]. Interestingly, we observed a gas plasma-mediated upregulation of HSF1 up to 24 h after gas plasma treatment in dermal fibroblasts, while one of its transcriptional targets, heat shock protein 90α (*HSP90α*), was reduced in pDFs after gas plasma treatment (Fig. S1b). These results suggested gas plasma treatment to dynamically affect integrin-adhesion complexes, mainly focal complexes (FXs) and focal adhesions (FAs).

Gas plasma treatment altered the composition and assembly of adhesion sites and enhances migratory properties during wound healing.

**Fig. 3:**
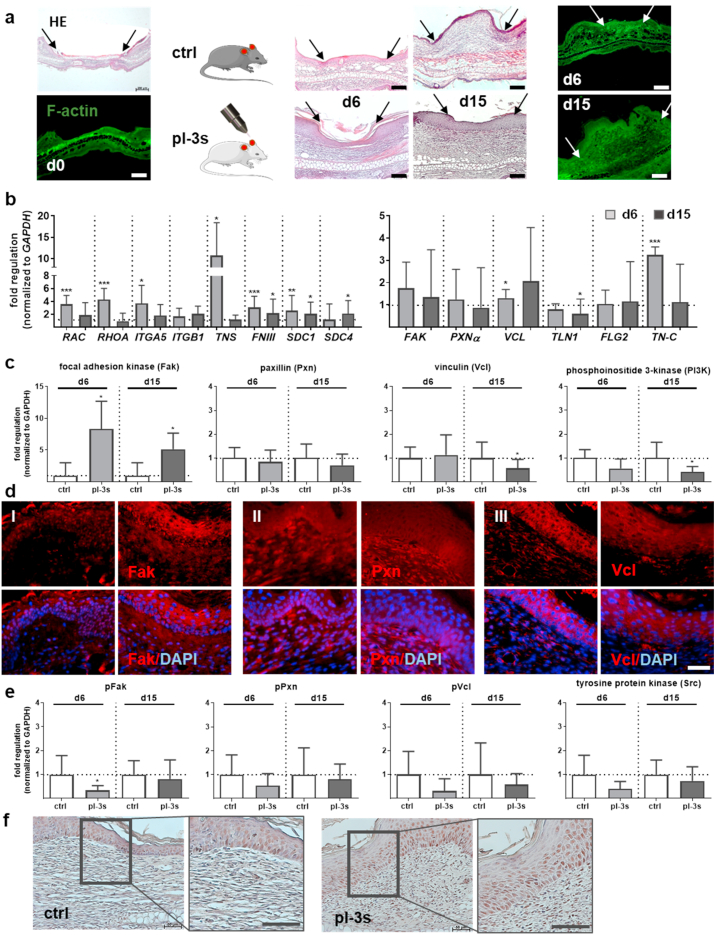
Gas plasma treatment altered the composition and assembly of adhesion sites and enhanced migratory properties during wound healing. (a) Ear wound sof SKH1 mice were either exposed to gas plasma (pl-3 s) or left untreated (ctrl) for 6 or 15 days. Representative HE staining of PFA-fixed sections showing a regular wound closure with early inflammation (d6), followed by re-epithelialization and tissue granulation in the proliferative phase (d15). FITC-phalloidin (green) was used to visualize the actin network on d0, d6, and d15, and the wound area was marked with arrows. (b) Quantitative mRNA expression analyses of *RAC, RHOA, ITGA5, ITGB1, TNS, FNIII, co-receptors SDC1/4, FAK, PXNα, VCL, TLN1*, and *TN-C* were performed. (c) Focal adhesion kinase (Fak), paxillin α (Pxnα), vinculin (Vcl), and phosphoinositide 3-kinase (PI3K) expression was quantified using WES analysis on d6 and d15. (d) Immunostaining of Fak (I), Pxnα (II), and Vcl (III) in controls (left panels) and gas plasma-treated wounds (right panels) on d6. (e) Western blot analyses of phosphorylated pFak, pPxn, pVcl, and tyrosine protein kinase (Src). (f) Phosphorylated Fak expression and its distribution were measured by pY397-FAK immunoreactivity on d15 (brown cells). Scale bars are 100 μm (a, f), 50 μm (d), 20 μm (f; right images). Data were normalized to *GAPDH* and untreated controls (ctrl) and presented as mean +SE. Statistical analysis was done by unpaired, two-tailed *Student*'s t-test (n > 8) with *p < 0.05, **p < 0.01, and ***p < 0.001. (For interpretation of the references to color in this figure legend, the reader is referred to the Web version of this article.)

We next attempted to validate the above-mentioned *in vitro* findings in gas plasma-treated murine wounds of full-dermal thickness wound healing model in SKH1 mice. H&E staining of the progression of wound closure as well as F-actin staining showed no significant differences between gas plasma-treated (pl-3 s) and untreated control (ctrl) mice (Fig. 3a). In terms of target expression and contrasting the *in vitro* results, we found a significantly higher expression of both members of the Rho GTPase family *RAC* and *RHOA* in gas plasma-treated mice on d6 but not d15 (Fig. 3b). Expression of integrin *ITGA5* but not *ITGB1* was strongly increased.

The presence of FAs and their dynamic development into FBs *in vivo* was supported by the notion that *TNS*, which plays an active role in FN fibrillogenesis, was highly upregulated (up to 10-fold) in response to gas plasma on d6. Fibronectin (*FNIII*), a multidomain protein with the ability to bind simultaneously to many FN molecules (e.g., cell surface receptors, collagen, proteoglycans) [10], was also significantly upregulated in comparison to untreated wounds. Similar to our *in vitro* findings in pDFs, both growth factor co-receptors (*SDC1/4*) were significantly upregulated on both early and late time points of gas plasma-assisted wound healing (left diagram, Fig. 3b), indicative of increased cell migration and growth factor uptake (Fig. S2a).

We also observed an increase in *VCL* and Tenascin C (*TN-C*, a glycoprotein expressed in the ECM) and a decrease in *TLN1* (a ligand of vinculin) levels in gas plasma-treated wounds on d6 in comparison to untreated controls. Again matching the *in vitro* findings, *PXNα* showed no gas plasma treatment-related changes in our skin tissue. *In vivo*, this was also the case for filaggrin (*FLG2*), a filament-aggregating protein, while it was upregulated *in vitro* in gas plasma-treated keratinocytes (Fig. S1b). Similar to cultured pDFs, Fak expression was elevated in gas plasma-treated wounds on the protein level (Fig. 3c) and when investigated using immunofluorescence in the epidermal layers of skin tissue (Fig. 3dI). Although we found a similar protein expression pattern and distribution of non-activated Pxnα (Fig. 3c/dII), the unphosphorylated paxillin was predominantly found in the dermal fibroblast containing skin layers and, to a lesser extent, in the epidermis (Fig. 3c/dII). As a functional consequence, gas plasma-induced changes of FAs such as Vcl revealed a significantly down-regulation on d15, as shown by Western blot analysis and immunofluorescence (Fig. 3c-dIII), similar to PI3K.

Next, we examined the expression of activated (phosphorylated) proteins. However, the levels of phosphorylated Vcl and PI3Kwere decreased by trend, similar to those found with Pxn (Fig. 3e). We obtained a roughly similar immunoreactivity for pY397-FAK in the epidermal and dermal layers of gas plasma-treated skin wounds compared to untreated wounds on the endpoint of measurement d15 (Fig. 3f). These data suggested regulation of the number, size, and distribution of adhesion complexes during wound healing following gas plasma exposure.

Gas plasma treatment affected fibroblast-to-myofibroblast and epithelial-to-mesenchymal transition.

**Fig. 4:**
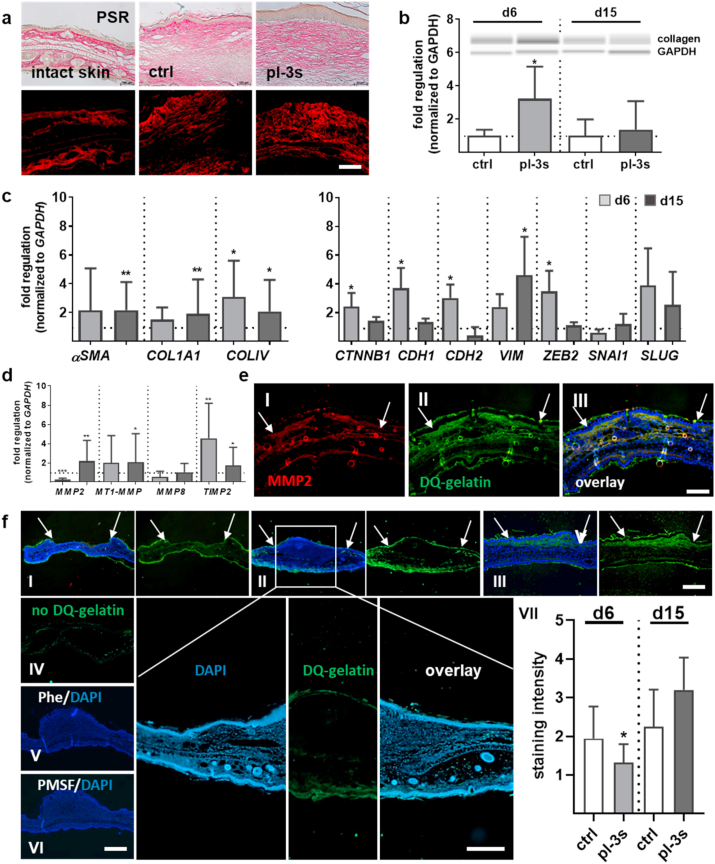
Plasma-driven cellular transformation and assessment of gelatinolytic activity during wound healing. (a) PSR staining of collagen fibers in gas plasma-treated wound tissue compared to untreated control showing granulation tissue with well-organized collagen fibers in parallel bundles. (b) Quantification of collagen expression levels as determined by Western blot analysis. (c) *αSMA*, *COL1A1*, and *COLV* gene expression levels were determined after 6 and 15 d following gas plasma treatment using qPCR and corresponding primers. Expression of characteristic EMT markers including β1-catenin (*CTNNB1*), epithelial E-cadherin (*CDH1*), mesenchymal N-cadherin (*CDH2*), vimentin (*VIM*), melanocytic transcription factor zinc finger E-box-binding homeobox 2 (*ZEB2*), and the transcription factors SNAI1 and SLUG after 6 d and 15 d following gas plasma treatment using qPCR and corresponding primers. (d) qPCR of *MMP2*, *MT1-MMP*, and their inhibitor *TIMP2* using qPCR. (e) Co-localization of MMP2 activity (red, I) and gelatinase (green, II) using immunostaining with anti-MMP2 primary and Alexa 594 secondary antibodies (III, merge). (f) The gelatinolytic activity of MMP2/9 was determined using *in situ* zymography utilizing DQ gelatin (green) in PFA-sections. Representative images of gelatinolytic activity on d0 (I), d6 (II, and higher magnification with scale bar = 200 μm), and d15 (III). *In situ* zymography without DQ gelatin (green autofluorescence, IV), with a metalloproteinase inhibitor 1,10-phenanthroline (Phe, V), and a serine protease inhibitor PMSF (VII). The gelatinolytic activity of gelatinases was quantified in controls vs. gas plasma-treated samples on d6 and d15 by scoring of the signaling intensity between 1 and 10 (VII). The cell nuclei were counterstained with DAPI (blue), and the wound area was marked with arrows. Scale bars are 50 μm (a), 100 μm (e). Data were normalized to *GAPDH* and untreated controls (ctrl) and presented as mean +SE. Statistical analysis was done by unpaired, two-tailed *Student*'s t-test (n > 4) with *p < 0.05, **p < 0.01, and ***p < 0.001. (For interpretation of the references to color in this figure legend, the reader is referred to the Web version of this article.)

Resident fibroblasts increase reactively in response to the microenvironment's external stimulation by secretion of excessive ECM components like collagens. Histological staining with PSR was conducted to determine the gas plasma-effects on the organization of collagen fibers and protein expression. Although collagen fibers' orientation was similar, we found small, parallel bundles along with a greater collagen density and fiber size in the connective tissue of gas plasma-treated mice (Fig. 4a). The quantification of collagen by Western blot showed a substantial increase of this structural ECM protein on d6. Its expression decreased to control levels on d15 (Fig. 4b), suggesting typical scar formation in which dermal ECM composition and structure were altered dependent on the wound healing phase only. The mRNA expression level of collagens *COL1A1* and *COLIV* were significantly increased on both time points compared with control groups (Fig. 4c). A comparison of collagen levels in pDFs showed that *COL1A* expression was strongly enhanced, whereas the expression of *COLIV* was up- and down-regulated concerning treatment and incubation time (Fig. S2b). Due to alpha-smooth muscle actin (αSMA) in stress fibers and microfilament bundles, cells exhibit contractile properties, which play a significant role in contraction during wound closure. The αSMA expression level was higher in gas plasma-treated wounds (Fig. 4c) and fibroblasts (Fig. S2b) at all time points investigated, showing that the differentiation of active fibroblasts into myofibroblasts (FMT) was vital in re-epithelialization from d6 to d15. The fibroblasts' ability for an efficient FMT upon gas plasma treatment with strong incorporation of αSma in cytoskeletal filaments was validated by immunofluorescence labeling (Fig. S2c).

Moreover, cells alter their gene expression to facilitate cellular changes such as epithelial-to-mesenchymal transition (EMT) in cutaneous wound healing [37]. To define this process, we have examined the expression levels of epithelial (e.g., β1-catenin and E-cadherin) and mesenchymal markers (e.g., N-cadherin, vimentin) by qPCR. A mesenchymal state is associated with increased expression of vimentin and N-cadherin under the transcriptional regulation of the zinc finger protein *SNAI1*, which was significantly increased in pDFs suggesting gas plasma-promoted repression of the adhesion molecule E-cadherin (*CDH1*) (Fig. S2d). Our findings further validated that wound tissue exhibited different states along the EMT spectrum. *CTNNB1*, *CDH1*, and *CDH2* were significantly upregulated on d6, whereas the *VIM* expression level was significantly increased on d15. EMT is regulated by distinct actions of several transcription factors [38], whereas only *ZEB2* and *SLUG* (*SNAI2*) but not *SNAI1* as EMT-promoting factors were gas plasma-regulated in our study (Fig. 4c).

Additionally, modulators of actin dynamics and F-actin networks are regulatory proteins and biomarkers of cellular protrusions. They consist of lamellipodia, filopodia, and podosomes and promote diverse processes of cell migration [33]. Interestingly, we found that these modulators, such as cortactin (*CTTN),* were significantly upregulated in the early stages of wound healing (d6), but not on d15. CTTN is concentrated at cytosolic actin, where it binds the Arp 2/3 complex. Similarly, several members of this actin-related protein complex (*ARPC1a, 3, 4, 5*) and the Wiskott-Aldrich syndrome (WAS)-related proteins (*WASp, WAS, WASI*) were enhanced upon gas plasma treatment on d6 (Fig. S2e), suggesting cellular protrusion formation with dynamic regulation of an actin assembly/disassembly supported cell migration. Overall, these results suggested a gas plasma effect on the fibroblasts' transition from an adherent state, characterized by focal adhesions and stress fibers, to a spreading cell phenotype with an absence of long actin stress fibers.

### 3.1 Distinct effects of gas plasma on extracellular matrix composition

Matrix metalloproteinases (MMPs) degrade matrix proteins and play essential roles in angiogenesis, tissue remodeling, and inflammatory responses to wounding [39]. The majority of MMPs are secreted as inactive proteases. Using protein lysates from homogenized wound tissues, we quantified expression levels of several MMPs and their inhibitors. A gas plasma-dependent regulation was found for *MMP2*, *MT1-MMP,* and *TIMP2* (Fig. 4d). The decrease of *MMP2* on d6 matched findings in pDFs at 2 h following gas plasma exposure, which showed an increase in TIMP2 also (Fig. S3a). In gas plasma-treated compared to control wounds, *MMP8*, *MMP9*, and *TIMP1* mRNA levels were similar (data not shown). DQ-gelatin labeling of tissue sections co-localized with MMP2, a gelatinolytic proteinase, confirming the presence of MMP2 in wounds (Fig. 4e). DQ-gelatin also allows quantifying the gelatinolytic activity of both gelatinases MMP2/9 by *in situ* zymography (Fig. 4f). On d0, gelatinolytic activity was found only in the non-wounded regions (**I**), while signal intensities in the wound regions increased on d6 (**II**) and d15 (**III**), which could not be attributed to autofluorescence (**IV**). The gelatinase inhibitors Phe (**V**) and PMSF (**VI**) abrogated DQ-gelatin fluorescence, indicating the assay's specificity. These findings suggested a dynamic regulation of gelatinase activity as a function of wound age. Quantification of gas plasma-treated wound sections showed a significant decrease of gelatinolytic activity on d6 compared to untreated wounds (**VII**). In supernatants of pDFs and using gelatin zymography, we discriminated between active and latent gelatinase forms and observed an increased secretion of active MMP2 and MMP9 24 h after gas plasma treatment (Fig. S3b).

### 3.2 Gas plasma treatment altered tissue oxygenation and microcirculatory parameters in wounds

**Fig. 5:**
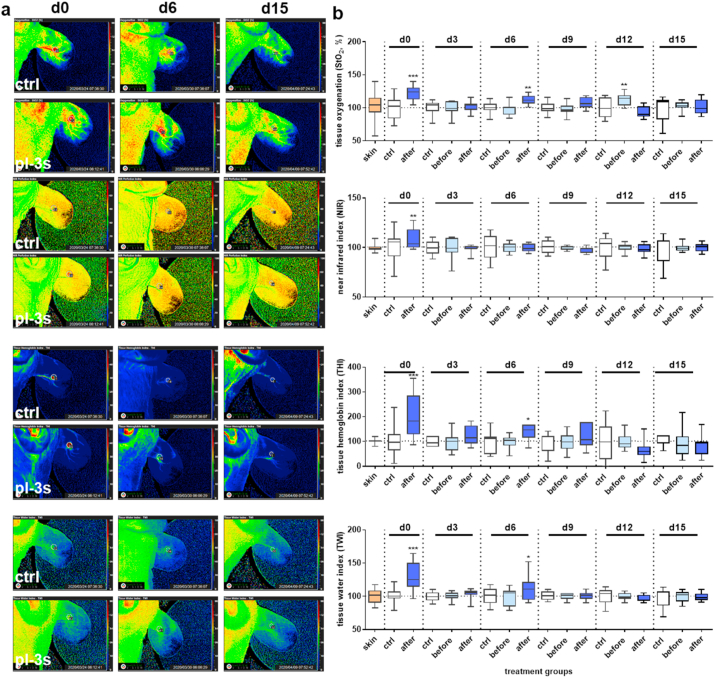
Microcirculatory measurements during wound healing. (a) Ear wounds were generated in a dermal full-thickness mouse model, gas plasma-treated (pl-3 s), and compared to controls (ctrl). Representative false-color images for each microcirculatory parameter on d0, d6, and d15. (b) Microcirculatory wound healing parameters were observed over 15 days using the TIVITA hyperspectral imaging camera system. The tissue oxygenation (StO2 in %), perfusion (NIR), tissue hemoglobin index (THI), and tissue water index (TWI) were measured every 3rd day after wounding in control mice and compared to unwounded tissue (orange). Quantification of characteristic spectral values for each microcirculatory parameter from 500 to 1000 nm after gas plasma treatment (light blue: before; blue: after gas plasma treatment) compared to untreated controls (white). Statistical analysis was performed using one-way ANOVA between gas plasma-treated and untreated mice (n = 4) with *p < 0.05, **p < 0.01, ***p < 0.001. (For interpretation of the references to color in this figure legend, the reader is referred to the Web version of this article.)

To determine physiological wound parameters on a macroscopic scale, we measured the tissue oxygenation levels (StO_2_) of the blood microcirculatory system in superficial tissue layers (1 mm penetration depth), the perfusion (in 4–6 mm depth) using near-infrared (NIR) index, the tissue hemoglobin index (THI), and the tissue water index (TWI). The assessment of these parameters was done every third day after wounding using the hyperspectral imaging system TIVITA. Initial measurements were done in untreated controls to compare the baselines of all four parameters across all groups, showing the changes in microcirculatory parameters compared to pre-operative conditions on d0 of unwounded tissue (skin, set to 100%, orange). For the wounding without gas plasma intervention, we found a slight increase of StO_2_ (Fig. S4a), whereas the other three parameters fluctuated around the baseline (Fig. S4b-d). Next, we quantified the spectral signals for each microcirculatory parameter at the corresponding wavelengths in the range from 500 to 1000 nm before (light blue) and after gas plasma treatment (3 s, blue) in comparison to untreated controls (white box plots). Analysis of parameters after wounding and gas plasma treatment revealed a significant increase in d0 and d6 (exception for NIR at d6) compared to untreated controls (Fig. 5a) as illustrated (Fig. 5b). No gas plasma-related changes were identified at later time points. Collectively, gas plasma treatment improved tissue oxygenation and hemoglobin and water indices at early wound stages.

## 4 Discussion

Gas plasma treatment was recently demonstrated to support wound healing in experimental models [19,27] and patients suffering from defective healing [24]. However, the mechanisms of action of this multi-ROS-producing technology in wounds are unexplored. The event of wounding and wound healing involves changes in the structure and composition of the extracellular matrix (ECM), cell signaling and migration, focal adhesion complexes, and proteolytic enzymes. Using various targets and analytical techniques, we identified gas plasma treatment to regulate all these processes, ultimately supporting wound healing.

**Fig. 6:**
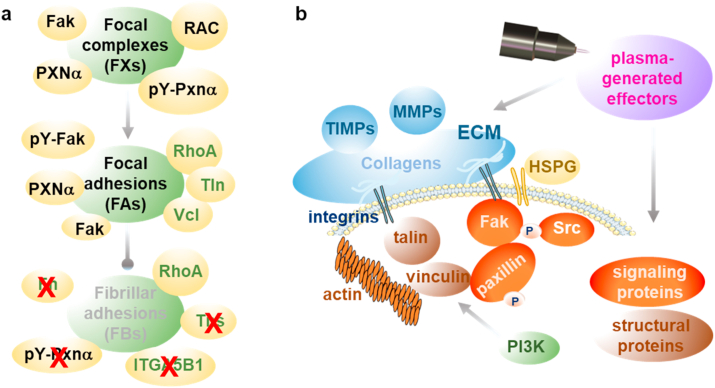
**Model of action of gas plasma treatment acting on structural and signaling proteins of integrin adhesion complexes.** (a*) In vitro*: gas plasma treatment of dermal fibroblasts affects integrin-adhesions indicating an absence of fibrillary adhesion (FBs) and its components (red cross) together with down-regulation of integrin α5β1, tensin (Tns), fibronectin (Fn), and RhoA (bright lettering). Gas plasma treatment further caused down-regulation of vinculin (Vcl) and talin (Tln) with increased expression of RAC, phosphorylated proteins such as focal adhesion kinase (Fak), and paxillin α (Pxnα), indicating the presence of focal complexes (FXs) and focal adhesions (FAs). Repeated gas plasma treatment of wounds in a murine model revealed a dynamic regulation of all three types of adhesion complexes with a corresponding expression signature (see also text). (b) Under physiological conditions, gas plasma-generated ROS function as signaling molecules and regulate cell adhesion and migration. The transmembrane heparan sulfate proteoglycans (HSPG), like syndecans (e.g., SDC4), cooperate with integrins (Itgα5β1) to regulate adhesion. Extracellular matrix proteins (e.g., MMPs and TIMPs), integrins, and HSPGs sense extracellular signals from gas plasma treatment by modifying the activation state of structural proteins (e.g., vinculin and talin), which are associated with actin and integrins. Such alteration is then transferred to signaling proteins by changing protein kinase phosphorylation levels (e.g., Fak, Src, and PI3K) as critical determinants of cellular responses. This leads to increased cell migration towards the wound bed, resulting in increased re-epithelialization and matrix deposition. Similar alterations and modulation of gene expression are initiated by the binding of growth factors to their respective receptors, emphasizing the crosstalk between growth factor-mediated and angiogenetic signaling. MMP, matrix metalloproteinase; TIMP, inhibitor of matrix metalloproteinase; Src, tyrosine kinase; PI3K, phosphoinositide 3-kinase. (For interpretation of the references to color in this figure legend, the reader is referred to the Web version of this article.)

A pivotal involvement of gas plasma in cellular migration as a sequence of repetitive steps in wound closure was identified. Such stimulatory effects on the migratory ability were also observed using single cell type and coculture models with cell lines upon gas plasma exposure *in vitro* models [40,41]. The present study demonstrates that a gas plasma-driven migration and spreading of skin cells was accompanied by a substantial change in gene expression of associated signaling pathways. The modulation of cellular adhesion to ECM is essential for cell motility and migration [[42], [43], [44]]. Cells contact ECM through various matrix contact structures such as integrin-adhesion complexes consisting of dynamically regulated focal complexes (FXs) as well as focal (FAs) and fibrillary adhesions (FBs) [45]. Signaling molecules are associated with integrin-mediated adhesions, such as paxillin and focal adhesion kinase, together with activation of small GTPases such as Rac and RhoA [46], eventually affecting integrin adhesion and its dynamics. In gas plasma-treated primary dermal murine fibroblasts, we demonstrated a down-regulation of Rac, the fibronectin (FN) receptor integrin α5β1, and tensin being a marker of FBs [8]. Moreover, a significant upregulation of Rho and tyrosine phosphorylation of paxillin was observed, indicating the presence of FXs and FAs but not FBs in gas plasma-treated fibroblasts. By contrast, the development of FAs into fibrillary adhesions (FB) is associated with the expression of tensin [8] and the integrins α5 and β1, which are localized in large paxillin-containing FBs of fibroblasts [47], which were downregulated in gas plasma-treated fibroblasts supporting the fact of an absence of FBs (Fig. 6).

Focal adhesions re-form at the cell-matrix contact points, where bundles of actin filaments are anchored to the integrin family's transmembrane receptors through a multi-molecular complex of junctional plaque proteins [1]. Notably, the concept of targeting integrins and associated complexes in the clinic has been established to treat cancers and other diseases [48], laying the basis for similar exploitation of integrins as targets for clinical gas plasma use in treating chronic wounds. As recently shown by proteome and gene profiling in gas plasma-treated HaCaT keratinocytes *in vitro* [49,50], a decrease in integrin expression often is associated with a loss of focal adhesion, a reduced cell adhesiveness, and increased cell motility [27]. Moreover, several studies have documented the differential expression patterns and functions of individual integrins in skin cells during wound repair [48], explaining the diversity of expression levels of single integrins in our study.

Many ECM and cytoskeletal genes showed higher expression upon gas plasma treatment, suggesting that ROS are strongly involved in their transcriptional regulation, for example, via Nrf2 signaling [51] and HSF1 signaling [36]. Nrf2, as the gatekeeper of intracellular and extracellular redox signals, counteracted oxidative stress via the upregulation of distinct downstream targets [19,27,49] and was extensively reviewed in Refs. [52]. The heat shock protein response (HSPs) via activation through cellular stress is mainly regulated by the translocation and phosphorylation of the transcription factor heat shock factor 1 (HSF1) [36]. However, HSF1 binds many non-HSP genes [53], which are essential in cell growth [54]. Here, we observed a plasma-driven upregulation of HSF1 in plasma-treated fibroblasts, which may have caused the observed transcriptional down-regulation of vinculin, an actin-binding adhesion molecule for cytoskeletal anchoring to cellular membranes [36]. Vinculin is associated with membrane-associated FA complexes and adherence junctions and links actin filaments to the ECM through talin and several integrins [32]. When fibroblasts were gas plasma-treated, vinculin switched to an inactive state resulting in the release from adhesion complexes, eventually enabling disassembly and disruption of the formation of the adhesive structures, allowing for increased cell migration. This result was supported by the fact that talin, a binding partner of vinculin, was down-regulated, whereas the anti-adhesive molecule tenascin-C was highly upregulated in fibroblasts. Tenascin-C is regulated by several growth factors, MMPs, and integrins during tissue remodeling [55]. Its ability to bind to the ECM glycoprotein fibronectin and to block fibronectin's interactions with specific syndecans might explain its anti-adhesive properties [56]. However, vinculin is known to increase during the change of cellular architecture by oxidative stress [57]. Cells expressing the active state of vinculin stabilize talin and exhibit increased adhesive strength [58], shown in early wound stages and gas plasma treatment *in vivo*. Vinculin-associated alterations result in the modification of intracellular signaling [13], intercellular communication [59], and regulation of the cell's motility, traits being essential for wound healing [60]. Therefore, we hypothesize that vinculin is a gas plasma-regulated switch in focal adhesion dynamics suggesting that vinculin is a useful target for optimizing care of non-healing wounds.

Integrin signaling is dependent on the non-receptor tyrosine kinase and the adaptor protein function of Fak for initiating downstream signaling events [61]. Our findings suggest an alteration of integrin adhesion complexes in gas plasma-treated fibroblasts and wounds in line with current knowledge on wound closure processes known to involve regulating phosphorylation of FAK and paxillin α (Pxnα). Tyrosine phosphorylation of paxillin induces cell spreading [62], migration [34], and assembly of focal complexes with focal adhesions [8], being is essential for stimulating the protrusive structures lamellipodia, filopodia, and podosomes [33]. Cellular protrusions are F-actin-rich matrix-degrading structures of migrating and invading cells at points of cell-matrix contacts. These protrusions were found in highly motile cells of mesenchymal lineages [33] and cell types subjected to stress [63]. We here document a gas plasma-driven modulation of cellular protrusions by spatial and temporal regulation of various protrusion markers (e.g., WASp, ARPC, and CTTN).

Several other molecules also govern the cellular filopodia and lamellipodia's dynamics and regulatory activities with specific roles in these structures, such as the membrane-associated MT1-MMP and F-actin [33]. First, matrix metalloproteinases (MMPs) determine cellular responses such as fibroblast proliferation, increased cell migration towards the wound bed, and matrix remodeling in the dermis. During the early stages of wound healing, the wounds become abundant in fibroblasts, which produce matrix proteins. The main enzymes for protein degradation (e.g., collagen) in the ECM are MMPs and their inhibitors (TIMPs) [39], which replace the fibrin clot with granulation tissue. We identified a wound stage-dependent gelatinase activity as an indicator of matrix degradation levels during gas plasma-spurred wound healing. Secondly, the formation of such structures is mainly triggered by actin polymerization and reorganization of actin cytoskeleton [64]. The re-epithelialization process is significant to the wound's contraction, which is aided by fibroblasts and myofibroblasts in the dermis with contractile abilities [65].

Gas plasma treatment was previously linked to the production of ECM components [66] and the differentiation of dermal fibroblasts toward a more contractile myofibroblast phenotype expressing αSma [20,67]. In our study, gas plasma-treated primary fibroblasts also exhibited a strong incorporation of αSma in cytoskeletal filaments suggesting an increase of contractile properties with fewer and less robust actin stress fibers. Similarly, fibronectin fibrillogenesis was impaired in gas plasma-treated fibroblasts due to the phosphorylation of paxillin [9]. This result suggested that gas plasma affected not only Fak and Pxn signaling (culminating in the reorganization of the actin cytoskeleton, a prerequisite for changes in cell shape, motility, and gene expression) but also fibronectin (FN) fibrillogenesis. Typically, FN fibrillogenesis is initiated by cytoskeleton-derived tensional forces transmitted across transmembrane integrins within the FN type III domains [68].

The binding of growth factors to their respective receptors also modulates gene expression. Gas plasma exposure induced uptake of these factors with a differential expression of syndecans 1 and 4. These cell surface proteoglycans are involved in transmembrane signaling and act as co-receptors together with other classes of cell surface molecules. The extracellular domain of syndecans can be cleaved from the cell surface [69], converting the membrane-bound proteoglycan into a paracrine effector molecule with essential roles in tissue repair [70]. This emphasizes the considerable crosstalk between adhesion and growth factor-mediated signaling in gas plasma-treated cells. The cooperative regulation of growth factors (e.g., VEGFA, FGF, and TGFβ) is the most potent switch in wound angiogenesis [71].

Angiogenesis is a dynamic process that is highly regulated by signals from the surrounding ECM environment and occurs in the wound tissue to increase the repairing tissue's metabolic needs. Previous studies have shown that gas plasma-treated wounds display increased wound closure with faster re-epithelialization and regrowth of the dermal layers. The responsiveness to gas plasma-mediated beneficial effects also relates to Nrf2 signaling together with increased vascularity and angiogenesis at the wound sites [19,26,27]. Here, we demonstrated a pro-angiogenetic nature of gas plasma, which was validated by finding increased tissue oxygenation in superficial skin layers after gas plasma treatment. Sufficient oxygen levels allow adequate epithelialization and granulation formation being a requisite for wound healing [72].

A major question in the field of gas plasma medicine is which type(s) of ROS/RNS are responsible for the effects observed [73]. We have recently demonstrated that gas plasma treatment directly oxidizes biomolecules of the target tissue, e.g. lipids [74]. At the same time, many of the chemical redox reaction pathways in gas plasmas have been unraveled [75], also for the jet system used in this study [21]. For instance, gas plasma-generated hydroxyl radicals and superoxide deteriorate to hydrogen peroxide [76] entering cells through aquaporins [77] and being intertwined with inflammatory redox signaling processes [78]. The kINPen plasma jet also generates reactive nitrogen species such as nitric oxide and peroxynitrite [79,80] that are both agents known to elicit specific physiological and immune-related effects as well as posttranslational protein modifications (PTMs), respectively [81,82]. Moreover, the jet generates singlet delta oxygen [83] and atomic oxygen [84], which together with chloride can give rise to hypochlorous acid [85] and subsequent cellular responses and immunogenic PTMs [86,87]. Limitations in techniques and model system do not allow yet disentangling the extent of each single type of ROS/RNS hitting and modifying the target cells and their proteins and lipids. By modulating the gas composition fed into the kINPen plasma jets, the biomedical consequences of distinct ROS/RNS compositions can be compared to each other [88]. Using this model system, we previously provided evidence of an enhanced tumor toxic potential *in vivo* using a gas plasma condition rich in atomic oxygen [89]. By applying a similar approach to other gas plasma applications in dermatology in the future, further light will be shed on understanding the mechanism of actions as well as improved plasma sources design and engineering for tackling dermatology-related diseases [90].

## 5 Conclusion

Substantial gene expression changes accompanied gas plasma-driven migration and the spreading of skin cells together with dynamic regulation of integrin adhesions. These results were supported by finding i) phosphorylation of the focal adhesion kinase and paxillin, ii) alterations of members of the GTPase family (e.g., RhoA, Rac), structural proteins (e.g., vinculin, talin), and actin organization, and iii) formation of new cell-matrix contacts. Gas plasma-derived ROS promote the physical integrity of healed skin, presumably via modulation of collagen deposition, the extracellular matrix degradation, and a wound healing stage-dependent regulation of proteinases (e.g., MMPs). This was attended by an enhanced oxygenation and tissue-hemoglobin perfusion of the superficial skin layer. Our model highlights the mechanisms of gas plasma treatment as a critical modulator of cell adhesion during wound healing via several molecular elements. These findings shed further light on targeting distinct cell populations (e.g., dermal fibroblasts) and novel therapeutic interventions using gas plasma technology to alleviate defective wound healing.

## Declaration of competing interest

The authors declare that they have no known competing financial interests or personal relationships that could have appeared to influence the work reported in this paper.

## Footnotes

Appendix A Supplementary data to this article can be found online at https://doi.org/10.1016/j.redox.2020.101809.
