# Supplementary material for: Gas plasma-spurred wound healing is accompanied by regulation of focal adhesion, matrix remodeling, and tissue oxygenation
Source: Redox Biol. 2020 Nov 25;38:101809. doi: 10.1016/j.redox.2020.101809 (PMC7710641; doi:10.1016/j.redox.2020.101809)
Supplement: Multimedia component 1 [file mmc1.docx]

Supplemental Data

Table S1. Murine gene-specific primers used in quantitative real-time PCR.

| Gene name | Gene ID | Primer sequences (3`- 5`) |
| --- | --- | --- |
| α smooth-muscle actin | *αSMA* | CCC AGA CAT CAG GGA GTA ATG G  TCT ATC GGA TAC TTC AGC GTC A |
| focal adhesion kinase | *FAK (PTK2)* | GAG TAC GTC CCT ATG GTG AAG G  CTC GAT CTC TCG ATG AGT GCT |
| Vinculin | *VCL* | GCT TCA GTC AGA CCC ATA CTC  AGG TAA GCA GTA GGT CAG ATG TG |
| collagen 1a1 | *COL1A1* | GCT CCT CTT AGG GGC CAC T  ATT GGG GAC CCT TAG GCC AT |
| collagen IV | *COLIV* | GGC CCC AAA GGT GTT GAT G  CAG GTA AGC CGT TAA ATC CAG G |
| paxillin α | *PXN*α | ACT TGA CCG GCT GTT ACT GG  GGG CTC GAT TCG GCT TCA T |
| talin 1 | *TLN1* | ATG TTA GAC GGA ACG GTG AAG A  CAA TTC GGG CAC AAA TGG TCA |
| fibronectin 1 | *FN1* | GCT CAG CAA ATC GTG CAG C  CTA GGT AGG TCC GTT CCC ACT |
| integrin B1 | *ITGB1* | TGG TCA GCA ACG CAT ATC TGG  GAT CCA CAA ACC GCA ACC T |
| integrin A1 | *ITGA1 (CD49a)* | GAC AGC CCT TGG AAT AGA CAC  GTT GTC ATG CGA TTCTCCATCA |
| integrin A2 | *ITGA2 (CD49b)* | TGT CTG GCG TAT AAT GTT GGC  TGC TGT ACT GAA TAC CCA AAC TG |
| integrin A5 | *ITGA5 (CD49e)* | TGC AGT GGT TCG GAG CAA C  TTT TCT GTG CGC CAG CTA TAC |
| integrin A6 | *ITGA6 (CD49f)* | GGG ATC GTC CGT GTA GAA CAA  TCT CTC CAC CAA CTT CAT AGG G |
| integrin AV | *ITGAV (CD51)* | AAA GAC CGT TGA GTA TGC TCC A  ATG CTG AAT CCT CCT TGA CAA AA |
| matrix metalloproteinase 2 | *MMP2* | GAC ATA CATCTTTGCAGGAGACAAG  TCT GCG ATG AGC TTA GGG AAA |
| matrix metalloproteinase 9 | *MMP9* | CCT GGA ACT CAC ACG ACA TCT TC  TGG AAA CTC ACA CGC CAG AA |
| matrix metalloproteinase 14 | *MMP14 (MT1-)* | ACC CAC ACA CAA CGC TCA C  GCC TGT CAC TTG TAA ACC ATA GA |
| MMP inhibitor 2 | *TIMP2* | GCC AAA GCA GTG AGC GAG AAG  CAC ACT GCT GAA GAG GGG GC |
| syndecan 1 | *SDC1* | AAC GGG CCT CAA CAG TCA G  CCG TGC GGA TGA GAT GTG A |
| syndecan 4 | *SDC4* | TTT GCC GTT TTC CTG ATC CTG  TTG CCC AAG TCG TAA CTG CC |
| heat shock protein 90α | *HSP90α* | GAC GCT CTG GAT AAA ATC CGT T  TGG GAA TGA GAT TGA TGT GCA G |
| β1 catenin | *CTNNB1* | CCC AGT CCT TCA CGC AAG AG  CAT CTA GCG TCT CAG GGA ACA |
| E-cadherin | *CDH1* | CAC CTG GAG AGA GGC CAT GT  TGG GAA ACA TGA GCA GCT CT |
| zinc finger E-box-binding 2 | *ZEB2* | ACC GCC GTC ATT TAT CCT GAG  CAT CTG GTG TTC CGT TTT CAT CA |
| zinc finger protein 1 | *SNAI1* | CAC ACG CTG CCT TGT GTC T  GGT CAG CAA AAG CAC GGT T |
| zinc finger protein 2 | *SLUG* | CAG CGA ACT GGA CAC ACA CA  ATA GGG CTG TAT GCT CCC GAG |
| vimentin | *VIM* | CGT CCA CAC GCA CCT ACA G  GGG GGA TGA GGA ATA GAG GCT |
| heat shock protein 8a | *HSP8A* | CGT ACC TCG GAA AGA CCG TTA  GTT GCC TGT CGC TGA GAG TC |
| Wiskott–Aldrich Syndrome protein | *WASp* | CAC CAG CAC CAA TCA ATG AGG  ACA AGT CCT AGA GAT AGT GGA CC |
| cortactin | *CTTN* | ATG GGG TGC TAA AAC CGT G  CTC CTT GAG CGT CTG GTG TTC |
| actin-related protein 2/3 complex | *ARPC2/3* | CCG GCA TAC CAC TCT TCT CTC  GGG CGG GTC CTT TGA ACT G |
| glyceraldehyde 3-phosphate dehydrogenase | *GAPDH* | CAT GGC CTC CAA GGA GTA AG  TGT GAG GGA GAT GCT CAG TG |


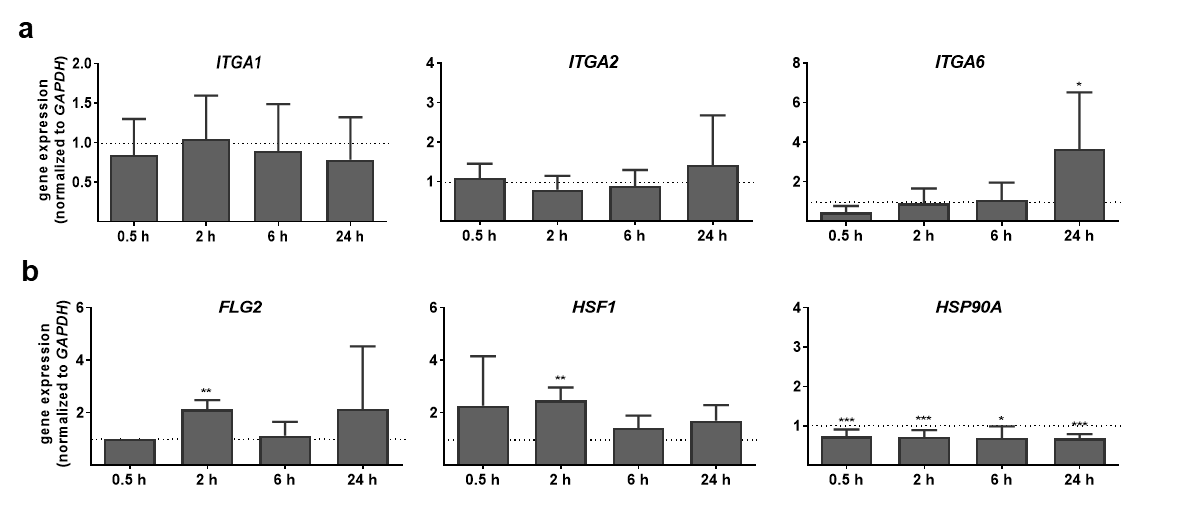


Figure S1. Gas plasma treatment altered integrin expression and ROS-related targets. (a) Gene expression levels of integrins (*ITGA1, ITGA2, ITGA6*) in pDFs. (b) Gene expression of filaggrin (*FLG2*) in gas plasma-treated keratinocytes, and of ROS-related targets such as heat shock factor 1 (*HSF1*) and *HSP90α* in pDFs. Data were normalized to *GAPDH* expression in untreated cells (ctr) and presented as mean + SE. Statistical analysis was done by unpaired, two-tailed *Student*’s t-test (n>4) with **p*<0.05, ***p*<0.01, and ****p*<0.001.


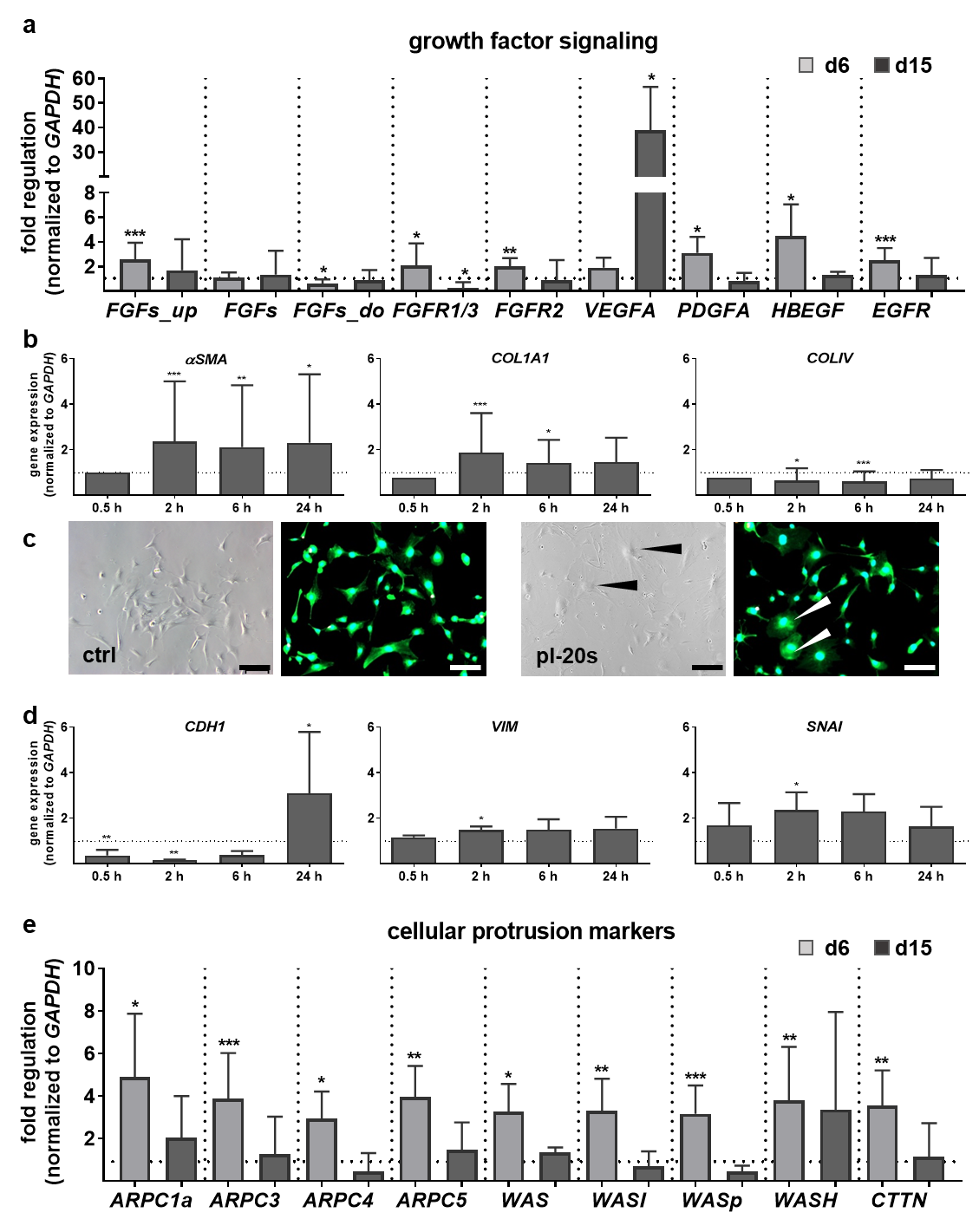


Figure S2. Gas plasma treatment altered signaling and scaffolding proteins that link the integrin receptors to the actin cytoskeleton. (a) Transcript analysis of distinct targets on early (d6) and late stages (d15) of wound healing showed a differential gene expression of growth factors and their receptors such as upregulated FGFs (FGFs_up: *FGF1/7/8/18*), non-regulated FGFs (*FGF9/10/15/21*), down-regulated FGFs (FGFs_do: *FGF3/6/13/23*), *FGFR1/3*, *FGFR2*, *VEGFA*, *PDGFA*, *HBEGF*, and *EGFR* *in vivo*. (b) Gene expression levels of smooth muscle actin (*αSMA*) and collagens (*COL1A1, COLIV*) in pDFs. (c) Light microscopy and immunofluorescence images of αSma-expressing myofibroblasts (green, arrowheads) 6 h after gas plasma treatment (pl-20 s). Scale bars are 100 µm. (d) Differential gene expression of the EMT markers *CDH1 and* *VIM,* and the transcription factor *SNAI1* in pDFs. (e) Transcript analysis of specific targets of cellular protrusions including the actin-related protein complex 2/3 (*ARPC1a*, *3*, *4*, and *5*), the Wiskott-Aldrich syndrome proteins (*WASp*, *WAS*, *WASI*, *WASH*), and cortactin (*CTTN*) using qPCR *in vivo*. Data were normalized to *GAPDH* and untreated controls (ctrl) at indicated time points (n>5) and presented as mean + SE. Statistical analysis was done by unpaired two-tailed *Student*'s t-test with significances of **p*<0.05, ***p*<0.01, and ****p*<0.001.


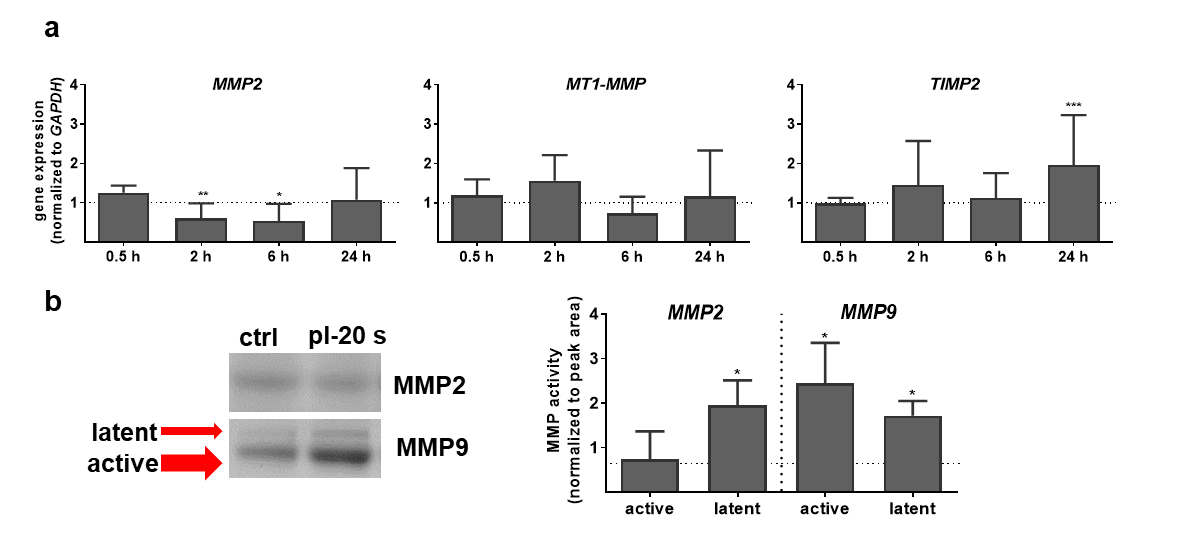


Figure S3. Gas plasma-driven cellular transformation and assessment of gelatinolytic activity during wound healing. (a) pDFs were indirectly incubated with gas plasma-treated serum-free medium for 24 h. qPCR of *MMP2*, MMP2-regulatory proteins MT1-MMP (*MMP14*), and *TIMP2* was performed. (b) For determination of gelatinase (secreted MMP2/9) activity in supernatants, active and latent forms of MMP2 and MMP9 were quantified and normalized to untreated cells using densitometry analysis. Data were normalized to *GAPDH* and untreated controls (ctrl) at indicated time points (n>5) and presented as mean + SE. Statistical analysis was done by unpaired two-tailed *Student*'s t-test with significances of **p*<0.05, ***p*<0.01, and ****p*<0.001.


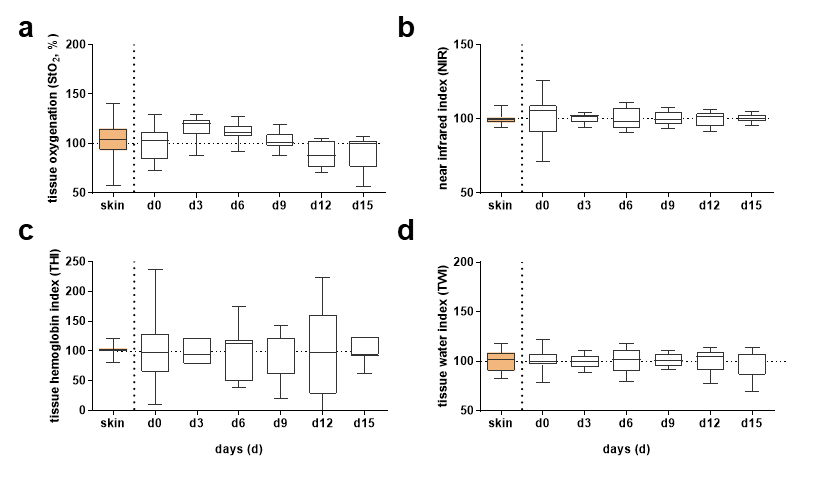


Figure S4. Microcirculatory measurements during wound healing. Ear wounds were generated in a dermal full-thickness mouse model. Microcirculatory wound healing parameters were observed over 15 days using the TIVITA hyperspectral imaging camera system. The tissue oxygenation (StO_2_, a), perfusion (NIR, b), tissue hemoglobin index (THI, c), and tissue water index (TWI, d) were measured every 3rd day after wounding in control mice and compared to unwounded tissue (orange).
